# Numerical Investigation of Reinforced Concrete (RC) Columns Strengthened with Ultra-High-Performance Fiber-Reinforced Concrete (UHPFRC) Jackets

**DOI:** 10.3390/ma17143380

**Published:** 2024-07-09

**Authors:** Andreas Lampropoulos, Spyridon Paschalis, Ourania Tsioulou, Stephanos Dritsos

**Affiliations:** 1 School of Architecture, Technology and Engineering, University of Brighton, Cockcroft Building, Lewes Road, Brighton BN2 4GJ, UK; tsioulou@hotmail.com; 2 School of Computing and Engineering, University of West London, Lady Byron Building, St Mary’s Road, London W5 5RF, UK; spyros.paschalis@uwl.ac.uk; 3 Department of Civil Engineering, University of Patras, 26500 Patras, Greece; dritsos@upatras.gr

**Keywords:** reinforced concrete, columns, strengthening, jackets, UHPFRC

## Abstract

The strengthening of existing columns using additional reinforced concrete (RC) jackets is one of the most popular techniques for the enhancement of a column’s stiffness, load-bearing capacity and ductility. Important parameters affecting the effectiveness of this method are the strength of the additional concrete, concrete shrinkage and the connection between the old and the new concrete. In this study, the application of Ultra-High-Performance Fiber-Reinforced Concrete (UHPFRC) jackets for the structural upgrade of RC columns has been examined. Extensive numerical studies have been conducted to evaluate the effect of parameters such as the thickness of the jacket, concrete shrinkage and the addition of steel bars, and comparisons have been made with conventional RC jackets. The results of this study indicate that the use of UHPFRC can considerably improve the strength and the stiffness of existing reinforced concrete columns. The combination of UHPFRC and steel bars in the jacket leads to the most effective strengthening technique as a significant enhancement in the stiffness and the ultimate load capacity has been achieved.

## 1. Introduction

The majority of the existing structures in earthquake prone areas need to be strengthened either because they have been damaged in previous earthquakes or because they have been designed without or with old code provisions. The use of additional reinforced concrete (RC) layers or jackets has been proved to be an effective technique and there are various published studies in this field [1,2,3,4,5,6,7,8,9,10,11,12,13,14,15,16,17,18]. These studies highlight the effectiveness of the use of RC elements and it has been found that two important parameters which affect the performance of the jacket using this technique are the connection between the old and the new concrete and the shrinkage of the new concrete of the jacket [16,17,18]. The use of steel jackets has also been proved to be effective for the enhancement of the strength and ductility of RC columns, which is attributed to the effect of the confinement [19]. In the last few decades, there has been an enormous development in the field of novel high-performance materials which have great potential for applications in the field of repairing and strengthening existing structures. The use of novel cementitious materials such as the polypara-phenylene-benzo-bisthiazole (PBO) fabric-reinforced cementitious matrix (FRCM) has also been studied for strengthening applications [20]. Extensive research has been conducted on the development and applications of Ultra-High-Performance Fiber-Reinforced Concrete (UHPFRC). UHPFRC is characterized by a significantly enhanced compressive strength which exceeds 150 MPa, a tensile strength normally higher than 7–8 MPa, and superior ductility and energy absorbance which are attributed to the high-strength cementitious matrix in addition to the high-volume fraction of steel fibers [21,22]. UHPFRC is characterized by significantly enhanced durability and low permeability, which are key factors for the protection of existing structures, while it has also been found that UHPFRC has excellent interlocking and bonding with existing concrete substrates when it is used as a repair material [23,24]. Extensive research has been conducted in this field and it has been found that the mechanical characteristics of UHPFRC are significantly affected by the volume fraction of steel fibers in addition to the orientation and distribution of the fibers [25,26,27].

There are research studies which have also focused on the use of alternative types of non-metal fiber reinforcement such as synthetic fibers, inorganic fibers, natural fibers and fibers from recycled glass [28,29,30,31,32,33]. The use of synthetic fibers normally leads to reduced strength compared to the respective steel fiber UHPFRC and this is attributed to the reduced fiber-to-cementitious matrix bond [28,31,32]. Also, in some cases, the use of ultra-high-molecular-weight polyethylene fibers has led to enhancements in the tensile strength but there is a reduction in the compressive strength. It has been proved that steel fibers are more effective than the use of macro fibers from recycled glass due to the increased elastic modulus of steel [33].

UHPFRC has great potential for the structural upgrade of existing structures; the majority of the existing studies in this field are focused on the strengthening of RC beams [34,35,36,37,38,39,40,41] and it has been proved that it can be effectively used for the structural strengthening of existing RC beams. UHPFRC layers reinforced with steel bars can offer superior structural performance and the connection between the old and the new interface is significantly enhanced, while the use of dowel can further improve the structural performance of the beams [36]. The addition of steel bars to the UHPFRC layers has been found to be able to lead to a significant enhancement in the load-bearing capacity up to 183%. Also, it has been found that the use of dowels at the interface leads to almost perfect connection between the existing structure and the UHPFRC layer, which results in an increment in the load-carrying capacity of around 12% for a 30 mm UHPFRC layer and 35% for a 70 mm UHPFRC layer [36]. UHPFRC has also been found to be quite effective for the prevention of bond failure in cases of deficient lap splices in beams and bridge columns. The application of UHPFRC strips for preventing shear failure has also been studied [41]. The use of UHPFRC has also been successfully applied for the retrofitting of a bridge [42] and for the strengthening of slabs, enhancing the energy absorption and the post-cracking performance of the existing elements [43].

The use of UHPFRC for the strengthening of existing RC columns is an area where further research is required as there are only very limited published studies [44,45,46]. The axial load-bearing capacity of jacketed columns with UHPFRC under eccentric loading has been studied [44], showing that the stiffness, the strength and the toughness of the strengthened columns are significantly enhanced. UHPFRC has also been used for the confinement of circular RC columns [45]. This study aims to provide an in-depth evaluation of the lateral performance of columns strengthened with UHPFRC. A parametric numerical study has been conducted to evaluate and quantify the effect of key parameters such as the thickness of the jacket, the presence of additional steel bars and the shrinkage strain of the jackets on the structural performance of the strengthened columns. This study also aims to critically evaluate the effectiveness of the examined strengthening technique through comparisons with the use of conventional RC jackets, highlighting the main benefits of the use of UHPFRC in strengthening applications.

## 2. Numerical Modeling of Columns Strengthened with RC Jackets

In this study, the numerical approach presented in Section 2 has been used for material modeling. The examined specimens are reinforced concrete (RC) columns strengthened with jackets. The geometry of the existing columns was selected to be in agreement with a previous study [17] where the strengthening of the existing columns with conventional RC jackets was examined.

**Figure 1:**
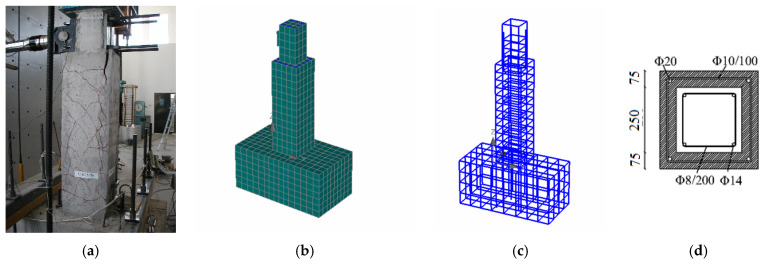
(**a**) RC-jacketed column tested in lab [14], (**b**,**c**) numerical models for concrete and for reinforcement, and (**d**) cross-section of strengthened column (dimensions in mm).

The initial column cross-section dimension was 250 × 250 mm and the height of the column was equal to 1800 mm, representing half of a full-scale column with the appropriate boundary conditions. The initial column was reinforced with four longitudinal steel bars with a 14 mm diameter and steel with a yield stress of 313 MPa and a rupture stress of 442 MPa. The same steel type was used for the 8 mm stirrups which were also placed along the height of the column at a spacing equal to 200 mm. A four-side RC jacket was used for the strengthening of the existing column with a thickness of 75 mm and a height equal to 1300 mm (Figure 1). The RC jacket is reinforced with four bars, 20 mm in diameter, with a yield stress of 487 MPa and rupture stress 657 MPa, and 10 mm diameter stirrups spaced at 100 mm with a yield stress of 599 MPa and a rupture stress of 677 MPa. Regarding the concrete, the initial column concrete compressive strength was found to be equal to 27 MPa, while the respective strength of the jacket was 55.8 MPa. Concrete compressive tests were conducted at the same time with the testing of the columns and after 28 days from the day of casting [12,13,15,17].

For numerical modeling, ATENA version 5 Finite Element Analysis (FEA) software was used [47]. Solid eight-node elements were used for the modeling of the concrete. Nonlinear behavior with softening in both tension and compression was considered. For compression, the CEB-FIP Model Code 1990 [48] model was used, while for tension, a linear ascending model followed by an exponential softening branch based on a fracture energy model was used [47].

The analysis was initially conducted assuming perfect connection between the old and the new concrete (specimen: monolithic).

For the simulation of the old-to-new concrete interface, special two-dimensional contact elements were used (specimen: R_INT._). To consider the strength degradation of the interface due to cycling loading, a reduction in the friction and cohesion characteristics with the loading cycles was proposed, which was found to lead to equivalent results with the use of coefficients of friction and cohesion equal to 1.0 and 0.0 MPa; therefore, these values were used in this study [17].

**Figure 2:**
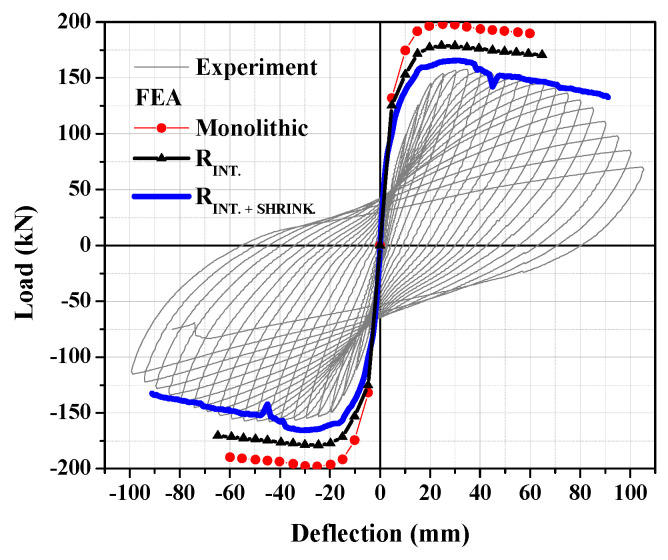
Experimental versus numerical results for strengthened elements with and without simulation of jacket’s concrete shrinkage.

To consider the effect of the jacket’s concrete shrinkage, a strain equal to 400 microstrains was also applied to the elements of the jacket, and in this numerical model, interface elements between the old and the new concrete were used with friction and cohesion coefficients equal to 1.0 and 0.0 MPa (specimen: R_INT.+SHRINK._). The numerical results of these three assumptions together with the respective experimental results are presented in Figure 2.

The results of Figure 2 show that the assumption of perfect bond at the interface (specimen: monolithic) leads to a significant overestimation of the structural behavior of the jacketed columns. In the case of specimen R_INT,_ where the old–new concrete interface is simulated without the presence of the jacket’s concrete shrinkage, the load capacity is reduced but there is still an overestimation of the structural performance. The numerical simulations of the strengthened columns with the interface with reduced friction and cohesion (to take into consideration the strength degradation) and with the simulation of the jacket’s concrete shrinkage (Specimen: R_INT.+SHRINK._) can accurately predict the response of the strengthened columns with RC jackets. The same assumptions have been used for the numerical simulations of the RC columns strengthened with UHPFRC jackets and the results are presented in Section 3.

## 3. Numerical Modeling of Columns Strengthened with UHPFRC Jackets

The characteristics of the initial RC columns are the same as the ones presented in Section 2. For the strengthening of the columns, UHPFRC jackets have been used with and without the presence of additional steel bars. The numerical assumptions for the modeling of UHPFRC are presented in Section 3.1.

### 3.1. Experimental Evaluation of UHPFRC Properties and Numerical Modeling

For the numerical simulation of UHPFRC, the compressive and direct tensile test results have been used. A typical UHPFRC mix design has been used in this study (Table 1 [35]). Regarding the mixing process, the dry materials were mixed first for 3 min followed by the addition of water and superplasticizer, while the steel fibers were added at the end of the process. The specimens were heat-cured at 90 °C for 3 days, and then, they were stored in ambient temperature and humidity conditions for 14 days until the time of testing.

**Figure 3:**
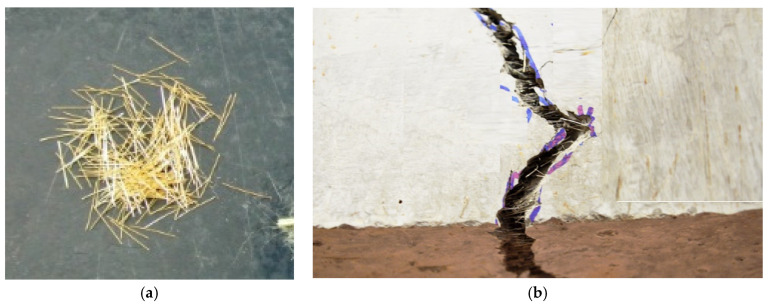
(**a**) Steel fibers used, and (**b**) typical fiber bridging in flexural testing of UHPFRC.

An illustration of the fibers used in the UHPFRC and typical bridge cracking of the fibers in the UHPFRC elements are presented in Figure 3a,b.

**Figure 4:**
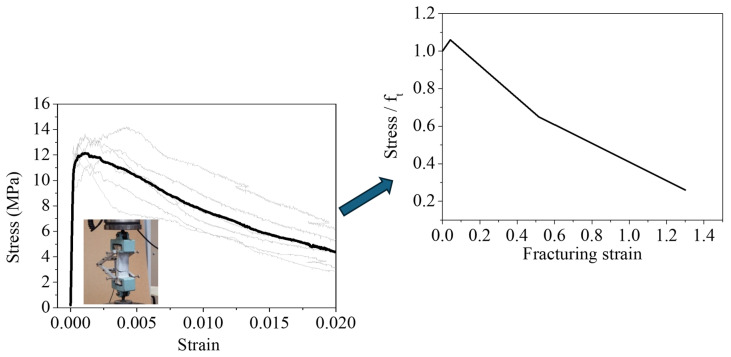
The experimental results of the direct tensile tests and the respective constitutive model [35].

For the evaluation of the compressive strength, standard 100 mm side cubes were tested according to the BS EN 12390-3 [49], while for the tensile stress–strain characteristics, dog-bone-shaped specimens were tested according to previous research in this field [35]. The compressive strength of UHPFRC was found to be equal to 164 MPa [35]. Six dog-bone-shaped specimens were also tested (Figure 4) to evaluate UHPFRC tensile stress–strain characteristics. UHPFRC is characterized by a significantly enhanced post-cracking tensile stress and superior energy performance, which can be accurately captured with direct tensile tests.

These tests have been performed under displacement control with a loading rate equal to 0.007 mm/s, and for the strain measurements, a Linear Variable Differential Transformer (LVDT) was used for the measurement of the extension over a length of 105 mm.

The stress–strain results for all of the examined specimens together with the averages are illustrated in Figure 4. ATENA version 5 FEA software [47] has been used for numerical simulations. The characteristics of the UHPFRC were determined from the compressive and tensile test results. The modulus of elasticity was taken as equal to 57.5 GPa and a compressive strength value of 164 MPa was used. The constitutive model for tension has been derived from the direct tensile test results and it consists of a linear part up to the tensile strength, which was calculated to be equal to 11.5 MPa, followed by a tri-linear part, as described in Figure 4. The fracturing strain values have been calculated considering the characteristic element size equal to 2 mm [35].

This modeling approach has been thoroughly examined in a previous research study where a systematic study on the calibration and validation of the numerical model for the simulation of UHPFRC elements using experimental data was presented [35].

**Figure 5:**
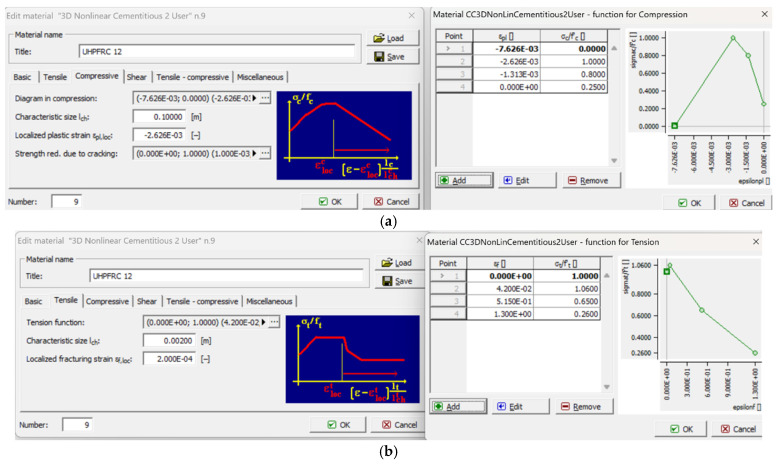
UHPFRC material model properties for (**a**) compression and (**b**) tension used for the numerical analyses of this study.

The characteristics of the material model used to simulate UHPFRC under compression and tension are illustrated in Figure 5a,b, respectively.

The results of the numerical investigation of RC columns strengthened with UHPFRC jackets are presented in Section 3.2.

### 3.2. RC Column Prior to and after Strengthening with UHPFRC Jackets

The initial pre-strengthened RC columns have the same characteristics as the ones described in Section 2, and the same modeling assumptions were used.

**Figure 6:**
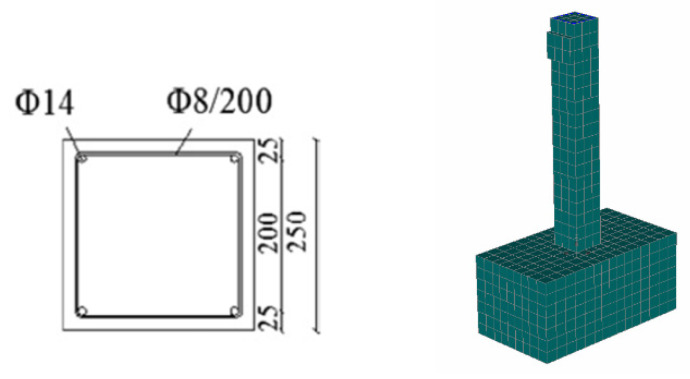
Numerical model and cross-section of initial RC column (dimensions in mm).

The numerical model geometry and mesh characteristics and the cross-section of the initial column are presented in Figure 6.

Regarding the modeling of the strengthened RC columns with UHPFRC jackets, the numerical assumptions of Section 2 were used. The old-to-new concrete interface was simulated with the same approach presented in Section 2 (contact elements with coefficients of friction and cohesion equal to 1.0 and 0.0 MPa, respectively).

An extensive parametric study was conducted to evaluate the effect of the jacket’s thickness and the effect of the shrinkage of the jacket on the performance of the strengthened columns; the results are presented in the following sections.

#### 3.2.1. Effect of UHPFRC Jacket Thickness

**Figure 7:**
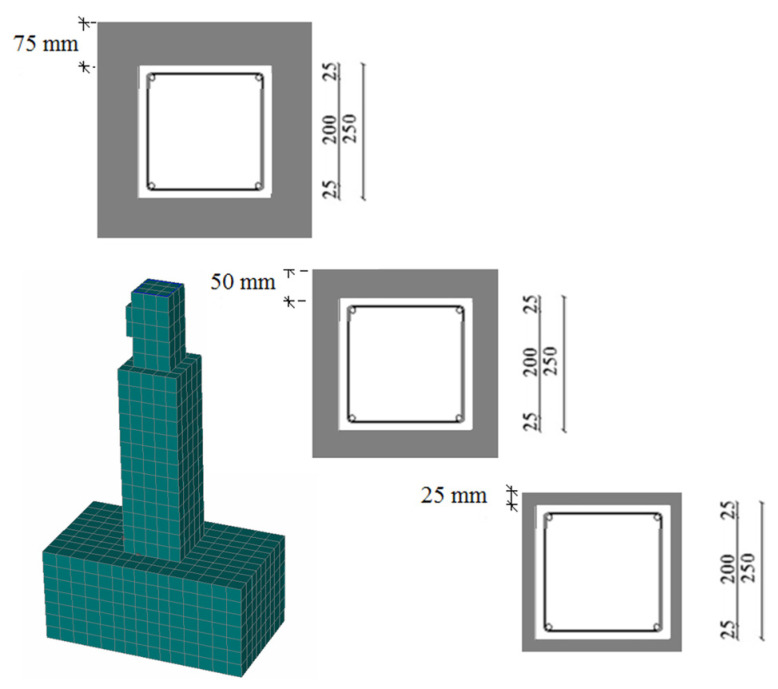
Numerical model and cross-sections of strengthened columns with UHPFRC jackets (dimensions in mm).

Three different thickness values were examined: 25 mm, 50 mm and 75 mm (Figure 7) [46]. For the shrinkage of the jacket, a value equal to 400 microstrains was applied, which represents an 800-microstrain free shrinkage value reduced to half to consider concrete creep [16].

**Figure 8:**
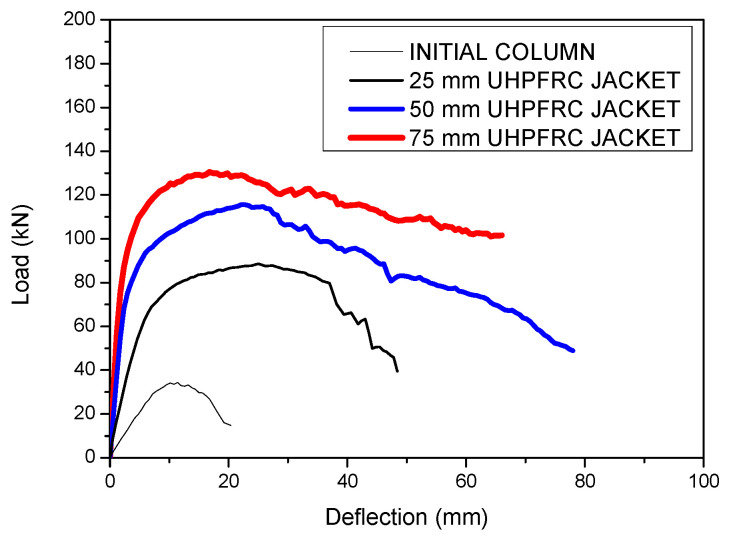
Load deflection results for initial and strengthened columns with UHPFRC jackets.

The results of the parametric study for the different values of the thickness of the UHPFRC jacket are illustrated in Figure 8 [46].

**Figure 9:**
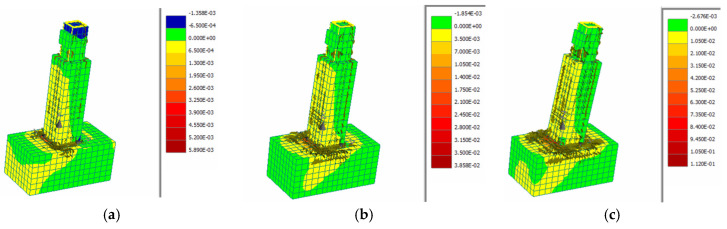
Strain and crack distribution for specimen with (**a**) 5 mm, (**b**) 20 mm and (**c**) 60 mm horizontal displacement.

The crack propagation and the strain distribution along the height of the column for the 75 mm UHPFRC jacket with 5 mm, 20 mm and 60 mm horizontal displacement at the top of the column are illustrated in Figure 9. These results show significant strain increments and subsequent crack development for 20 mm and 60 mm displacement, as expected.

**Figure 10:**
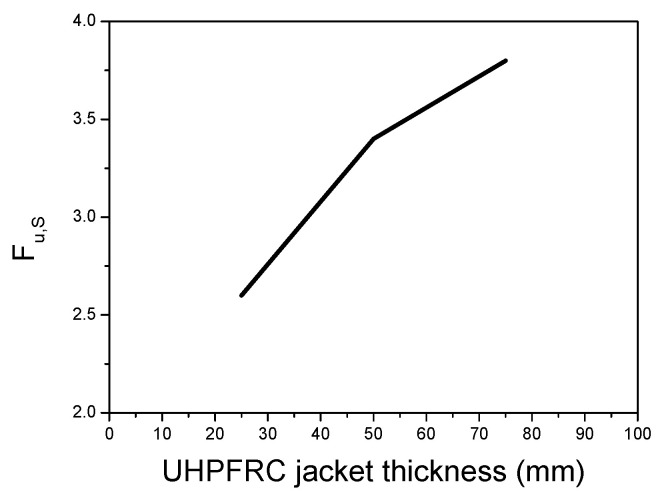
Strength enhancement ratios (***F_u,S_***) for different UHPFRC jacket thicknesses.

The results of Figure 8 show that the thickness of UHPFRC significantly affects the results, and as the thickness is increased, the stiffness and the ultimate load capacity are increased, as expected. The increment in the strength with the thickness of the UHPFRC jacket has been quantified using the ***F_u,S_*** ratio, which represents the ratio of the ultimate load of the strengthened columns over the respective results of the initial specimen (i.e., $F_{u,S}=\frac{F_{u,Strengthened}}{F_{u,Initial}}$). The results of ***F_u,S_*** for different values of thickness of the UHPFRC jacket are presented in Figure 10 [46].

The results of Figure 10 indicate that there is a significant effect of the thickness of the UHPFRC jacket on strength enhancement. An increment in the range of 2.6–3.8 times was observed for UHPFRC thicknesses of 25–75 mm.

#### 3.2.2. Effect of Addition of Steel-Reinforcing Bars in UHPFRC Jackets

An additional investigation has been conducted for the 75 mm thick UHPFRC jacket, where steel bars have also been used in addition to fiber reinforcement. Longitudinal bars and shear links have been used with the same characteristics as the ones used in the conventional RC jacket (Section 2, Figure 1c).

**Figure 11:**
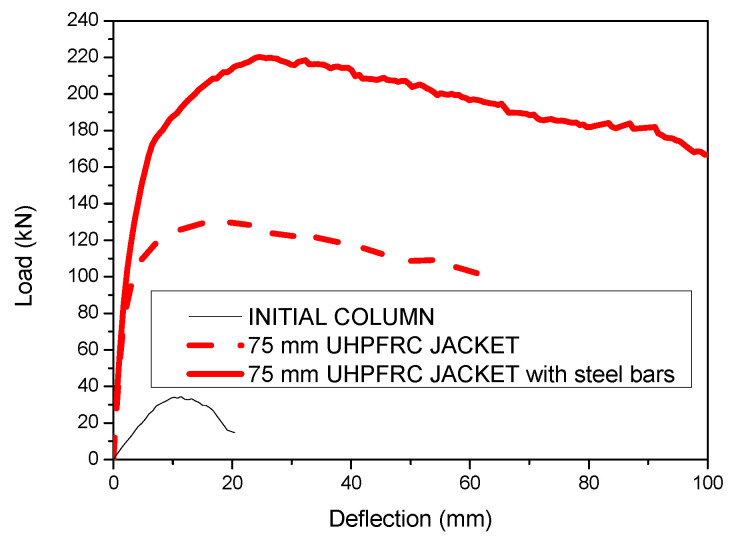
Load deflection results for initial and strengthened columns with 75 mm UHPFRC jacket with and without steel bars.

The results of the strengthened column with the 75 mm UHPFRC jacket with and without additional steel bars are presented in Figure 11.

The results of Figure 11 show that the addition of steel bar reinforcement leads to a significantly higher load capacity. More specifically, the ultimate load of the strengthened column with the 75 mm UHPFRC jacket is 131 kN, while with the addition of steel bars, this load is further increased by 68% and the ultimate load is equal to 220 kN.

#### 3.2.3. Effect of UHPFRC Jacket Shrinkage

**Figure 12:**
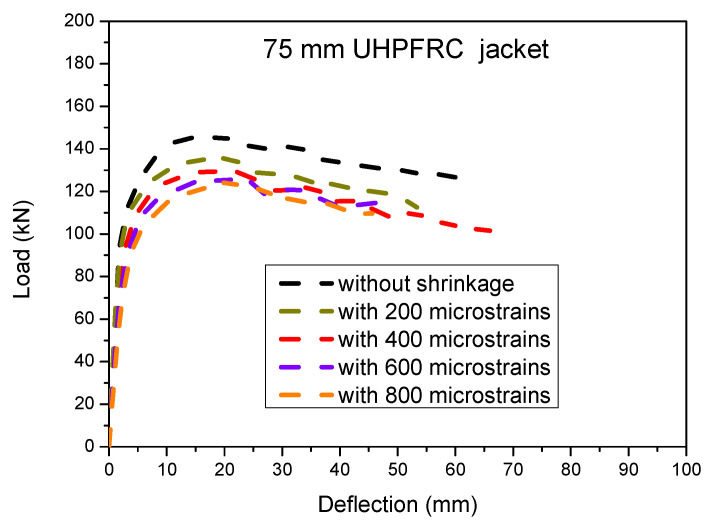
Load deflection results for 75 mm thick UHPFRC jacket using different UHPFRC shrinkage strain values.

The effect of the UHPFRC jacket’s shrinkage is presented in this section. A parametric study has been conducted for the 75 mm thick UHPFC jacket with different values of concrete shrinkage. More specifically, shrinkage strain values of 200, 400, 600 and 800 microstrains were applied to the UHPFRC jacket and the numerical analysis results are presented in Figure 12 [46].

**Figure 13:**
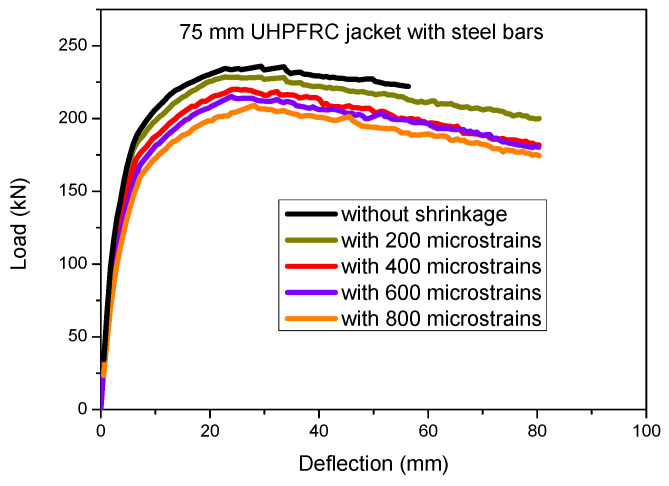
Load deflection results for 75 mm thick UHPFRC jacket with steel bars using different UHPFRC shrinkage strain values.

A parametric study to assess the variation in the UHPFRC shrinkage strain values has also been conducted for the case of the 75 mm UHPFRC thick jacket with additional steel bars and the results are presented in Figure 13.

The results of Figure 12 and Figure 13 show that there is a significant detrimental effect of the UHPFRC shrinkage strain on the ultimate load capacity of the strengthened columns. It can be observed that as the shrinkage strain values of the jacket are increased, there is a significant reduction in the maximum load capacity which is attributed to the induced tensile stresses due to the restrained concrete shrinkage of the jacket. This leads to a biaxial stress state which results in a reduction in the strength of the jacket and a subsequent reduction in the ultimate load capacity of the examined elements [17].

**Figure 14:**
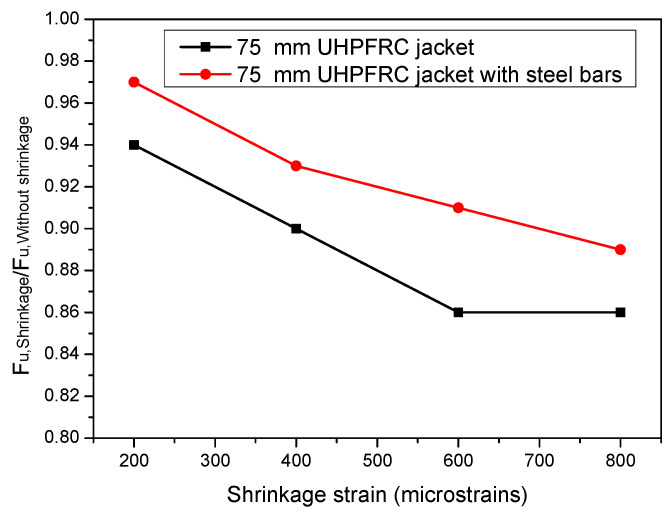
Variation in ***F_u,Shrinkage_***/***F_u,Without shrinkage_*** for various shrinkage strain values for jacketed columns with 75 mm thick UHPFRC jacket with and without steel bars.

The reduction in the ultimate load capacity has been quantified and the ratios of the ultimate load with and without shrinkage (***F_u,Shrinkage_***/***F_u,Without shrinkage_***) have been calculated for both cases of jacket columns with the 75 mm thick UHPFRC thick jacket with and without the additional steel bars; the results are presented in Figure 14.

The results of Figure 14 show that in case of the 75 mm thick UHPFRC jacket without steel bars, a reduction in the ultimate load of almost 15% was observed for shrinkage strain, equal to 800 microstrains. The detrimental effect of concrete shrinkage is limited by the presence of the steel bars of the jackets, as in the case of the jacketed column with the 75 mm thick UHPFRC jacket with additional steel bars, higher values of the ratio ***F_u,Shrinkage_/F_u,Without shrinkage_*** were derived compared to the respective values of the jacketed column with the 75 mm UHPFRC jacket without steel bars.

## 4. An Evaluation of the Effectiveness of the Use of UHPFRC Jackets and Comparisons with the Use of Conventional RC Jackets

**Figure 15:**
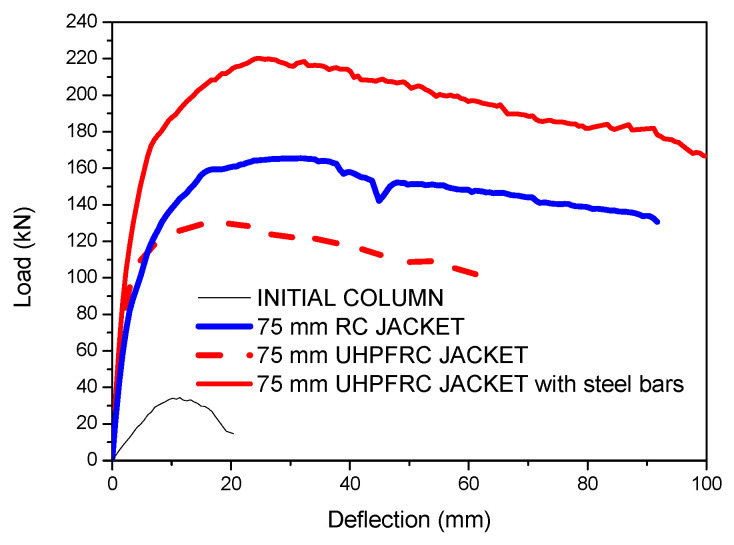
Load deflection results for initial column and for different strengthening techniques.

In this section, a critical evaluation of the results of the jacketed columns with conventional RC jackets, UHPFRC jackets and UHPFRC jackets with additional steel bars is presented. In all of the examined cases, a 75 mm thick jacket was used and a shrinkage strain equal to 400 microstrains was applied to the elements of the jacket. The load deflection results for all of these cases together with the results of the initial column are presented in Figure 15.

**Figure 16:**
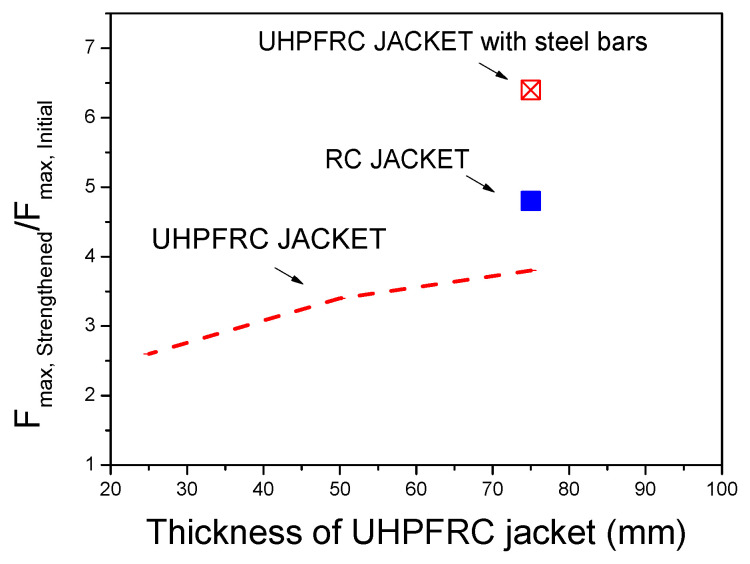
Ultimate load increments in UHPFRC- and RC-jacketed columns.

The ratio of the ultimate load capacity of the strengthened columns to the respective results of the initial column (***F_u,Strengthened_***/***F_u,Initial_***) for all of the different examined techniques is presented in Figure 16.

The results of Figure 15 show that with all of the strengthening techniques, the stiffness and strength are significantly increased compared to the respective results of the initial column. The addition of a UHPFRC jacket leads to greater stiffness enhancement compared to the respective results of the column strengthened with an RC jacket. The greatest stiffness and strength enhancement is achieved by the addition of a UHPFRC jacket with additional steel bars. From the comparisons of the ultimate load increment ratios (***F_u,Strengthened_***/***F_u,Initial_***) (Figure 16), it is evident that the increment in the ultimate load in the case of the UHPFRC jacket with steel bars is significantly higher than in all of the other techniques, as the ultimate load was found to be 6.4 times higher than the load of the initial column. The respective values for the RC jacket and for the UHPFRC jacket (without additional steel bars) were found to be equal to 4.8 and 3.8, respectively. These results highlight the significant contribution of the steel bars to the structural performance of the jacketed column, which, in combination with the UHPFRC, leads to the most effective strengthening method.

## 5. Conclusions

This study is focused on the effectiveness of the use of UHPFRC jackets for the structural strengthening of existing RC columns. It is the first time that the effect of key parameters such as the thickness of the jackets, the presence of steel reinforcing bars and the shrinkage of the UHPFRC jackets has been examined. The enhancement of the structural performance has been quantified for all of the examined cases, offering valuable information which could be used for design purposes and for the selection of the required characteristics of UHPFRC jackets. A critical comparison of this technique with the use of conventional RC jackets has also been conducted and the following conclusions were drawn.

▪ The thickness of the UHPFRC jacket significantly affects the stiffness and the ultimate load capacity, which increase as the jacket’s thickness is increased. The ultimate load capacity is increased by 2.6–3.8 times for UHPFRC thicknesses of 25 mm–75 mm compared to the respective load of the initial (prior to strengthening) RC column.
▪ The addition of steel bar reinforcement to the UHPFRC jackets leads to a significantly greater load capacity enhancement. In the case of the RC column strengthened with the 75 mm thick UHPFRC jacket, an ultimate load capacity of 131 kN was achieved and this value was further increased to 220 kN (68% increment) by the addition of steel bars to the UHPFRC jacket.
▪ UHPFRC jacket shrinkage leads to a reduction in the ultimate load capacity of the strengthened columns due to the development of tensile stresses in a direction normal to the loading condition and a subsequent biaxial stress state. A reduction in the ultimate load of almost 15% was observed for an imposed shrinkage strain of 800 microstrains. The detrimental effect of the concrete shrinkage is limited by the presence of steel-reinforcing bars in the UHPFRC jackets.
▪ The comparison of UHPFRC jackets with traditional RC jackets shows that the use of UHPFRC leads to greater stiffness enhancement compared to the respective results of the column strengthened with the RC jacket. The highest load enhancement was achieved for columns strengthened with the UHPFRC jacket with steel bars, where the ultimate load was found to be 6.4 times higher than the load of the initial column. In the case of the RC jacket and the UHPFRC jacket (without additional steel bars), the ultimate load increments were found to be equal to 4.8 and 3.8, respectively.

## Figures and Tables

**Table 1:** Mix design for UHPFRC [35].

Material	Mix Proportions (kg/m^3^)
Cement (52.5 N)	657
GGBS	418
Silica fume	119
Silica sand	1051
Superplasticizer	59
Water	185
3% Steel fibers (13 mm in length and 0.16 mm in diameter)	236

## Data Availability

The original contributions presented in the study are included in the article, further inquiries can be directed to the corresponding author.
